# Infective endocarditis complicated with multiple cerebral embolism and hemorrhage caused by *Streptococcus sinensis*: a rare case report and literature review

**DOI:** 10.3389/fmed.2026.1786870

**Published:** 2026-06-24

**Authors:** Hong Zheng, Cuilin He, Kai Tang, Xiwen Wang, Longbiao Xie, Wei Li, Jianli Wu, Danjie Zhao

**Affiliations:** 1 Department of Pharmacy, The First People’s Hospital of Shuangliu District, West China (Airport) Hospital of Sichuan University, Chengdu, China; 2 Department of Cardiovascular Medicine, The First People’s Hospital of Shuangliu District, West China (Airport) Hospital of Sichuan University, Chengdu, China; 3 Department of Clinical Laboratory, The First People’s Hospital of Shuangliu District, West China (Airport) Hospital of Sichuan University, Chengdu, China; 4 Department of Neurology, The First People’s Hospital of Shuangliu District, West China (Airport) Hospital of Sichuan University, Chengdu, China; 5 Department of Ultrasound Medicine, The First People’s Hospital of Shuangliu District, West China (Airport) Hospital of Sichuan University, Chengdu, China

**Keywords:** cerebral infarction, fever, infective endocarditis, intracerebral hemorrhage, *Streptococcus sinensis*

## Abstract

*Streptococcus sinensis* (*S. sinensis*) is a rare pathogen causing infective endocarditis (IE). We report a 68**-**year**-**old Chinese man with *S. sinensis-*associated IE complicated by cerebral infarction with hemorrhagic transformation. He presented with fever, aphasia, arthralgia, arthritis, and right hemiparesis. Initial transthoracic echocardiography was negative for vegetations; valve-adjacent vegetations were identified until after neurological stabilization. Antimicrobial therapy was adjusted iteratively according to culture and susceptibility results. Linezolid plus cefazolin for 4 weeks achieved his condition stabilization; the patient declined valve replacement and was discharged. Oral linezolid plus moxifloxacin was continued for another 4 weeks, with sustained stability. *S. sinensis* IE can involve the central nervous system. Early pathogen identification and targeted antimicrobial therapy are crucial for better outcomes.

## 1 Introduction

Infective endocarditis (IE) is a severe infection affecting the endocardial surface of the heart and cardiac valves, caused by bacteria, fungi, or other pathogens (including viruses and rickettsiae) that disseminated via the bloodstream. Although its annual incidence is relatively low, ranging from 3 to 9 cases per 100,000 population, IE remains the most destructive and fatal form of valvular heart disease (1). The most common causative pathogens include *Staphylococcus aureus*, *Viridans streptococci*, and *Enterococcus* species (2). *Streptococcus sinensis* (*S. sinensis*) belongs to the Mitis group of *Viridans streptococci* and was first isolated in 2002 from a 42**-**year**-**old Chinese woman with chronic rheumatic heart disease and IE-related mitral regurgitation. Named for its geographic origin (Hong Kong, China), this organism has since been reported in sporadic cases of IE in China, Italy, France, and the Netherlands, establishing it as a rare and emerging causative pathogen of IE (3).

The clinical diagnosis of IE remains challenging, particularly in patients with atypical initial manifestations or infections caused by rare pathogens. Conventional diagnosis relies on positive blood cultures, echocardiographic evidence of vegetations, and characteristic clinical features; however, early nonspecific symptoms frequently lead to delayed diagnosis. According to the 2023 European Society of Cardiology (ESC) guidelines (4) on IE, the most common clinical manifestations include fever (77%), cardiac murmurs (64.5%), and congestive heart failure (27.2%). Notably, neurological complications occur in 25.3% of patients, while 11.5% develop cardiac conduction abnormalities. Additional manifestations include peripheral erythema, splinter hemorrhages, Roth spots, and immune-mediated phenomena such as glomerulonephritis. Importantly, patients with IE who developed neurological complications exhibit nearly twofold increase of the 1**-**month mortality compared with those without such complications (5). Consequently, early surgical intervention is recommended when vegetations exceed 10 mm in size to prevent the occurrence of neurological complications. Epidemiological data indicate that *Staphylococcus aureus* and *Enterococcus* species are the predominant pathogens among IE patients with neurological complications. To date, only one case previously reported *S. sinensis-*associated IE complicated by neurological involvement. The patient died of multiple cerebral infarction after refusing surgical treatment, underscoring the diagnostic complexity and high mortality risk associated with this rare infection (6).

We report the case of a 68**-**year**-**old man who initially presented with fever, bilateral metatarsophalangeal joint swelling and pain, and lower-extremity edema. These symptoms highly resembled gouty arthritis, thereby obscured potential diagnosis of IE. Shortly after admission, the patient’s condition deteriorated rapidly, with the sequential development of extensive cerebral infarction and intracerebral hemorrhage. Although early blood cultures identified *S. sinensis*, initial transthoracic echocardiography failed to detect valvular vegetations, further complicating the diagnostic process. Sequential antimicrobial regimens, including levofloxacin plus penicillin and clindamycin plus cefazolin, produced suboptimal responses. After vegetations were subsequently detected on echocardiography, the treatment regimen was changed to linezolid combined with cefazolin, which ultimately resulted in clinical resolution. This case highlights a rare presentation of *S. sinensis*–induced IE complicated by multiple cerebral embolism with hemorrhagic transformation, providing critical clinical insights into the diagnosis and managements of IE caused by this uncommon pathogen.

## 2 Case presentation

**Figure 1:**
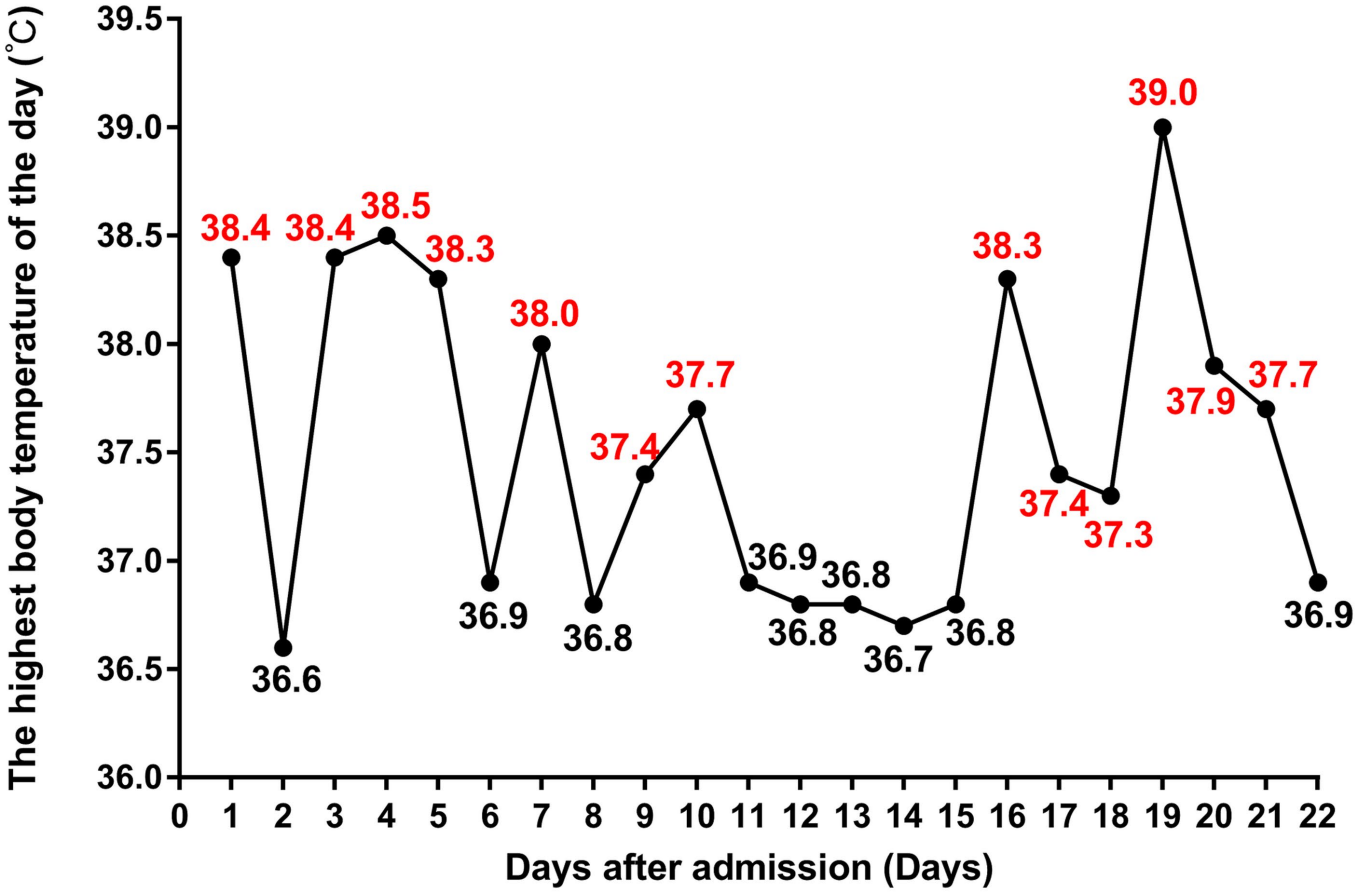
The maximum daily body temperature during hospitalization.

On June 5, 2025, a 68**-**year**-**old man was admitted with a 1**-**month history of recurrent bilateral metatarsophalangeal joint pain accompanied by local warmth and bilateral lower**-**extremity edema, followed by a 1**-**day fever. Prior to admission, the patient had self-medicated with unspecified drugs, resulting in transient symptom relief followed by worsening pain. The peak body temperature was 38.5 °C (Figure 1). His medical history was notable for previous varicose vein surgery.

On admission, vital signs were as follows: blood pressure: 139/54 mmHg, heart rate: 82 beats/min, respiratory rate: 20 breaths/min, temperature: 36.5 °C, and arterial oxygen saturation: 95.3%. Random fingertip blood glucose was 6.4 mmol/L and uric acid level was 320.9 μmol/L. The laboratory tests revealed a leukocyte count of 10.15 × 10^9/L, with neutrophilic granulocyte percentage at 78.9%. The C**-**reactive protein (CRP) was 76.22 mg/L, and procalcitonin was 0.37 ng/mL (Supplementary Table S1). Physical examination revealed intact consciousness, bilateral mild coarse breath sounds, mild bilateral lower**-**extremity edema, and tenderness, swelling, and localized skin warmth of both ankle joints and the first metatarsophalangeal joints. Chest computed tomography (CT) revealed a small nodular lesion in the posterior basal segment of the left lower lobe. The initial diagnosis was undifferentiated arthritis. In view of the accompanying cough and fever, pulmonary infection was also considered. Empirical antimicrobial therapy with cefoperazone**-**sulbactam (2 g intravenously every 12 h) was initiated, and paired blood cultures were obtained.

**Figure 2:**
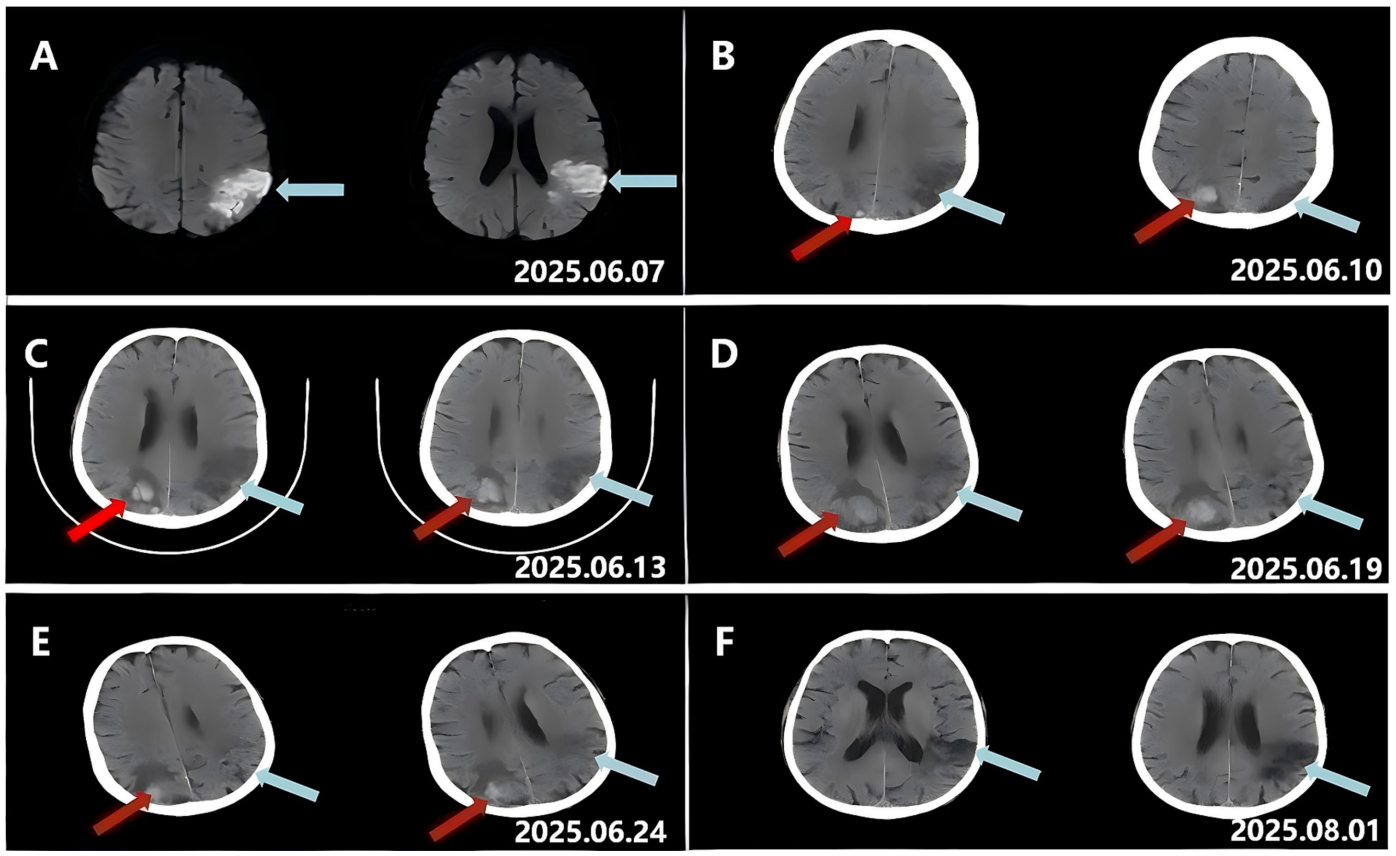
The magnetic resonance imaging or computed tomography results of brain. **(A)** Cranial magnetic resonance imaging at the initial occurrence of neurological complications. A large left-sided intracerebral infarction. **(B)** Computed tomography (CT) scanning with the brain on 5th day. A minor right-sided intracerebral bleeding and a stable left-sided intracerebral infarction. **(C)** CT scanning with the brain on 7th day. An increased right-sided intracerebral bleeding and a stable left-sided intracerebral infarction. **(D–F)** CT scanning with the brain on 15th, 20th, and 58th day. An improved right-sided intracerebral bleeding and a stable left-sided intracerebral infarction. Blue arrows indicate the location of the cerebral infarction. Red arrows indicate the location of the cerebral hemorrhage.

**Figure 3:**
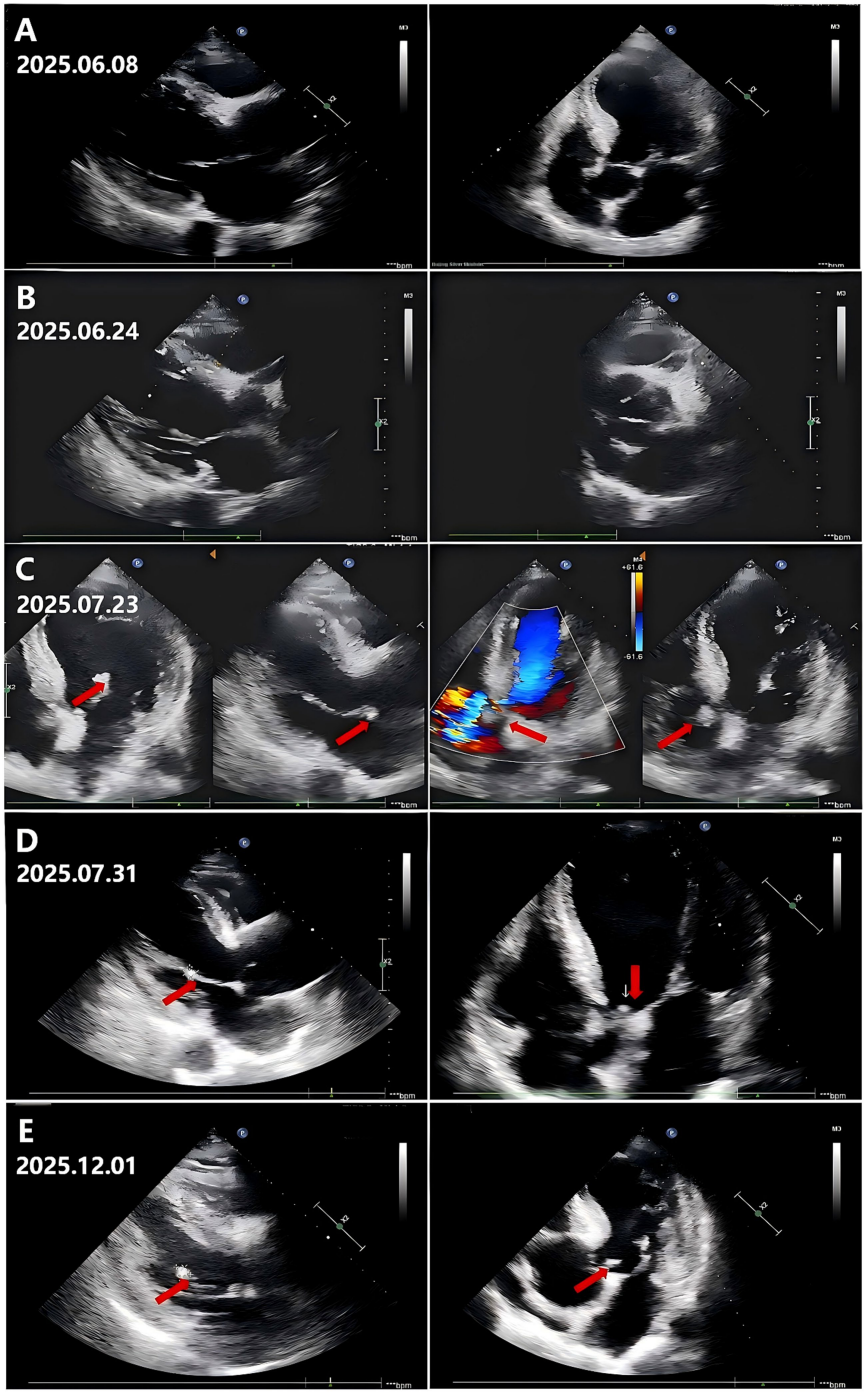
The transthoracic echocardiography results of the mitral and aortic valves. **(A)** No vegetations in the mitral valve (left ventricular long-axis view) and aortic valve (five-chamber view) on 5th day. **(B)** No vegetations in the mitral valve (left ventricular long-axis view) and aortic valve (short-axis view of the aorta) on 20th day. **(C)** Mitral valve vegetation 11 × 8 mm (left ventricular long-axis and four-chamber views) and aortic valve 18 × 15 mm (five-chamber view) vegetation on 49th day. **(D)** Mitral valve vegetation 10 × 7 mm (left ventricular long-axis view) and aortic valve 6 × 4 mm (five-chamber view) vegetation on 56th day. **(E)** Mitral valve vegetation 10 × 7 mm (left ventricular long-axis view) and aortic valve 7 × 5 mm (five-chamber view) vegetations on 86th day. Red arrows indicate the location of the vegetations.

On hospital day 1, the patient suddenly developed right**-**sided limb weakness, speech disorder and NIHSS score ≤3. Cranial CT revealed extensive acute infarction involving the left parieto**-**occipital region and insular cortex, multiple mixed plaques in the bilateral carotid arteries with mild to moderate luminal stenosis (Figure 2A). Neurological examination demonstrated impaired responsiveness, mixed aphasia, and right-sided hemiparesis, with muscle strength graded as 4/5 in the right limbs and 5/5 in the left. Following neurological consultation, dual antiplatelet therapy with aspirin (100 mg once daily) and clopidogrel (75 mg once daily), together with supportive treatment, was initiated. On hospital day 3, the patient again developed a fever (38.4 °C). As blood culture results were still pending, empirical antimicrobial therapy was escalated to meropenem (1 g intravenously every 8 h). On day 4, persistent fever (>38 °C) continued, and blood cultures identified *S. sinensis* (Supplementary Figure S1; Table 1). Bilateral ankle swelling and tenderness remained unchanged. Ultrasonography revealed a small effusion in the right ankle joint and tendinopathy of the right Achilles tendon. On day 5, the patient’s temperature normalized; however, his neurological status deteriorated, characterized by somnolence, mixed aphasia, facial asymmetry, and further decline in right**-**sided muscle strength (3+/5). Repeat cranial CT demonstrated new intracerebral hemorrhages in the right parietal and occipital lobes and the left cerebellar hemisphere (Figure 2B). All antiplatelet agents were immediately discontinued, while meropenem therapy was continued. Joint swelling and local hyperthermia persisted. A repeated cranial CT scan conducted 2 days later revealed an increase in the extent of hemorrhage in the right parietal and occipital lobes, as well as in the left cerebellar hemisphere, compared to the previous scan (Figure 2C). On day 10, neurological status improved, with restoration of consciousness and partial recovery of motor function, allowing ambulation. Joint pain and swelling were alleviated. Follow-up cranial CT showed decreased hematoma density and size, along with partial resolution of infarcted areas (Figure 2D). On hospital days 19**–**20, the patient experienced recurrent high**-**grade fever (up to 39 °C), despite marked improvement in joint symptoms and mobility. Repeat cranial CT demonstrated continued absorption of intracerebral hematomas with stable infarcted lesions (Figure 2E). Although two cardiac echocardiograms did not reveal any vegetations, except for moderate aortic regurgitation and left ventricular enlargement (Figures 3A,B), the recurrent fever, cerebrovascular events, heart failure, degenerative valvular disease, and the presence of cardiac murmurs strongly supported a diagnosis of IE based on multidisciplinary consultation. The patient was transferred to a tertiary hospital on day 21. Repeat echocardiography revealed thickened and roughened mitral valve leaflets with abnormal echogenic attachments on the valve and subvalvular chordae, consistent with vegetations. A definitive diagnosis of *S. sinensis-*associated IE was established. Based on antimicrobial susceptibility testing, sequential therapy with levofloxacin (0.5 g once daily) plus penicillin (2.4 g every 12 h) for 6 days, followed by clindamycin (1.2 g every 8 h) combined with cefazolin (1.5 g every 8 h) for 5 days, was administered. However, repeat echocardiography demonstrated enlargement of mitral valve vegetations to 10 × 7 mm and newly detected aortic valve vegetations measuring approximately 11 mm, indicating inadequate infection control. Antimicrobial therapy was therefore switched to linezolid (0.6 g every 12 h) combined with cefazolin (2 g every 8 h). After 7 days, follow-up imaging showed reduced intracranial hematoma volume and cerebral edema. Vegetation size stabilized to 11 × 8 mm on the aortic valve and 18 × 15 mm on the mitral valve (Figure 3C). Following clinical stabilization, the patient was transferred to our hospital on July 22, to continue intravenous linezolid and cefazolin therapy. During hospitalization, no further febrile episodes occurred, except for mild bilateral lower-extremity edema. Repeat paired blood cultures obtained on July 28 were negative. After 25 consecutive days of antimicrobial therapy, echocardiography showed significant reduction in vegetation size (Figure 3D), and cranial CT demonstrated stable infarction and hemorrhagic lesions (Figure 2E). Surgical removal of vegetations was recommended but the patient declined. He was discharged on August 3, and continued oral moxifloxacin (400 mg once daily) combined with linezolid (600 mg every 12 h) for an additional 4 weeks. Follow*-*up evaluation on August 30 showed no significant change in vegetation size, and antimicrobial therapy was discontinued (Figures 2F, 3E).

**Table 1:** Antibiotic sensitivity tests of *streptococcus sinensis* from blood culture.

Antimicrobial agent^a^	MIC (ug/mL)	Sensitivity	Determination standard		
			Sensitive	Intermediary	Resistance
Penicillin G	0.12	S	≤0.12	0.25–2	≥4
Tetracycline	≥8	R	≤2	4	≥8
Cefotaxime	≤0.5	S	≤1	2	≥4
Erythromycin	≥1	R	≤0.25	0.5	≥1
Telithromycin	≤1	–	–	–	–
Clindamycin	≥1	R	≤0.25	0.5	≥1
Quinupristin	≥4	R	≤1	2	≥4
Linezolid	≤2	S	≤2	–	–
Rifampicin	≤1	–	–	–	–
Levofloxacin	≤2	S	≤2	4	≥8
Moxifloxacin	≤1	–	–	–	–
Chloramphenicol	≤4	S	≤4	8	≥16
Vancomycin	≤1	S	≤1	-	-

^a^Tested by the broth microdilution method. MIC, Minimal inhibitory concentration; S, Sensitive; R, Resistance.

## 3 Pathogen identification and antimicrobial susceptibility testing

On June 5, two blood culture bottles (one aerobic and one anaerobic) were collected from a single venipuncture site. Both bottles became positive after 33.6 h of incubation, and pure colonies were obtained after 24 h of incubation on the blood lipid agar plate. The bacterial colony was identified as *S. sinensis* by matrix-assisted laser desorption/ionization time*-*of*-*flight mass spectrometry (MALDI*-*TOF MS, Zybio, EXS2600), with an identification score of 2.27 (Supplementary Figure S1). Antimicrobial susceptibility testing was performed using the broth microdilution method on the VITEK*-*2 Compact automated system (Table 1). The results were interpreted in accordance with the Clinical and Laboratory Standards Institute (CLSI) M100*-*S33 guidelines and U. S. Food and Drug Administration (FDA) criteria.

## 4 Review and discussion

In this case, the patient presented with fever complicated by cerebral infarction with hemorrhagic transformation. Early manifestations included joint pain, fever, and elevated nonspecific inflammatory markers, which initially led to a presumptive diagnosis of skin and soft*-*tissue infection. Subsequent blood cultures identified *S. sinensis*, raising suspicion of secondary sepsis originating from a soft*-*tissue source. Targeted antimicrobial therapy based on susceptibility testing resulted in transient symptomatic improvement; however, symptoms recurred within 2 days. Given the clinical course and the known propensity of *S. sinensis* to cause IE, *S. sinensis-*associated IE was strongly suspected. Nevertheless, the patient lacked classical features of IE, such as cardiac murmurs (no significant cardiac murmur was detected during auscultation in the acute phase of cerebral infarction), osler nodes or rheumatoid factor positive. Additionally, there were no apparent predisposing factors, such as prior cardiac surgery or recent dental procedures. Moreover, transthoracic echocardiography performed at the time of positive blood cultures did not reveal valvular vegetations, further increasing diagnostic uncertainty. IE was ultimately confirmed only after repeat echocardiography performed several days later demonstrated the presence of valvular vegetations. Among patients with IE complicated by neurological involvement, cerebral infarction with hemorrhagic transformation is an independent risk factor for mortality, with a reported incidence of approximately 22.7% (5). The most common causative pathogens in such cases were *Staphylococcus aureus* and *Enterococcus* species (5). Based on previously reported cases of *S. sinensis-*associated IE, the present patient represents the second documented case complicated by cerebral infarction with hemorrhagic transformation. Notably, the first reported patient died as a result of this complication (6). We hypothesize that valvular vegetations were initially small and therefore undetectable by transthoracic echocardiography. Under conditions of hemodynamic stress or heightened inflammation, these vegetations may have detached from the valvular surface and subsequently embolized to the cerebral circulation. Owing to the small caliber of cerebral vessels, embolic fragments can readily lodge within them, leading to vascular occlusion and subsequent cerebral ischemia, hypoxia, and tissue necrosis. This case underscores that IE should be considered a primary differential diagnosis in patients with fever of unknown origin accompanied by embolic phenomena*-*particularly cerebral embolism*-*and that transesophageal echocardiography (TEE) should be performed promptly to confirm the diagnosis. The 2023 guidelines (4) strongly recommend TEE in patients with high clinical suspicion of IE and negative transthoracic echocardiography, as TEE provides superior visualization of valvular structures.

The therapeutic course in this case was particularly challenging. As shown in Table 1, *S. sinensis* demonstrated *in vitro* susceptibility to multiple antimicrobial agents. Although treatment with meropenem for 20 days resulted in improvement of neurological symptoms, the patient continued to experience recurrent fever. Subsequent therapy with levofloxacin plus penicillin, followed by clindamycin combined with cefazolin for a total duration of 11 days, failed to control disease progression. From a pathophysiological perspective, heart valve damage exposes collagen, which becomes coated with platelets and fibrin, forming a non-bacterial thrombotic vegetation. Circulating bacteria (e.g., *Streptococcus*) bind to this exposed collagen via surface adhesins, triggering further platelet and fibrin deposition. Bacterial colonization factors (e.g., platelet bactericidal protein) then help bacteria embed within the vegetation, shielding them from shear stress and immune clearance. The result is a mature vegetation with a dense, metabolically dormant bacterial core that resists antimicrobial therapy. This can attenuate the efficacy of antimicrobial agents, leading to the persistent existence of large vegetations (7, 8). This can attenuate the efficacy of antimicrobial agents, leading to the persistent existence of large vegetations. During this period, continued enlargement of aortic and mitral valve vegetations was observed, suggesting inadequate infection control. Concurrently, the acute phase of intracerebral hemorrhage precluded surgical intervention, necessitating repeated adjustments of antimicrobial therapy to limit disease progression. Ultimately, treatment with linezolid combined with cefazolin stabilized vegetation size at 6 × 4 mm on the aortic valve and 10 × 7 mm on the mitral valve. Notably, approximately 60% of *Streptococci* do not directly trigger platelet aggregation, but initiate a two**-**stage process: they first undergo independent adhesion via binding to specific sites on platelets, and subsequently induce aggregation mediated by platelet aggregation-associated proteins. Experimental observations show that after aggregation induction, the morphology of the vegetations can change from flat to spherical or spiky (9). This mechanism can, to a certain extent, explain the clinical phenomenon that vegetations suddenly increase significantly after multiple negative transthoracic echocardiography results.

After stabilization of the intracerebral hemorrhage, the patient met clear surgical indications according to the European Society of Cardiology (ESC) guidelines for IE, including valvular dysfunction with a tendency toward heart failure, large and mobile vegetations, and a high risk of embolization (4). Previous studies have demonstrated that when vegetation size exceeds 30 mm, early and effective antimicrobial therapy alone does not significantly reduce the incidence of neurological complications, and early surgical intervention, comprising vegetation removal with valve replacement, remains the most effective strategy to reduce mortality and recurrence (10). However, the patient declined surgical treatment. Fortunately, compared with the previously reported fatal case, the vegetations in this patient were relatively small (approximately 10 mm). Follow-up at 1 and 3 months after discharge confirmed survival without evidence of infection recurrence. A review of previously reported cases of *S. sinensis-*associated IE reveals substantial heterogeneity in treatment strategies. Prior to 2022, isolates were generally susceptible to penicillin G, ceftriaxone, levofloxacin, and aminoglycosides, particularly *β-*lactam antibiotics, and standard regimens such as ampicillin or amoxicillin, with or without gentamicin for more than 4 weeks, were usually effective (3, 6, 11–16). However, reports published after 2022 describe cases in which similar susceptibility profiles were observed, yet patients experienced persistent fever and progressive vegetation enlargement despite these regimens, necessitating multiple antibiotic changes. In these cases, agents such as vancomycin, teicoplanin, and linezolid were frequently required, with treatment durations extended beyond 6 weeks (17–21). In the present case, switching to linezolid combined with cefazolin achieved effective infection control. This favorable response may be attributable to the initial involvement of the joints and central nervous system, as linezolid exhibits excellent tissue penetration, which may represent a key factor contributing to therapeutic success.

To date, *S. sinensis* has been reported exclusively as a cause of IE, with no documented infections involving other anatomical sites. Some studies have hypothesized that this organism possesses virulence factors that facilitate adhesion to and colonization of cardiac valves, thereby inducing endocardial injury (21). Among the 14 cases reported worldwide (Table 2), including the present case, patient ages ranged from 8 to 68 years, with our patient representing the oldest reported individual. Nine patients had congenital or acquired heart disease (3, 11–14, 17, 19, 20); three had undergone dental procedures (13, 19, 21); two required tooth extraction for severe dental caries (13, 14); one was receiving long-term corticosteroid therapy for giant cell arteritis (16); one underwent coronary angiography at disease onset (18); and one had chronic anemia with severe renal dysfunction (6). Clinical manifestations varied widely among reported cases: fever occurred in 11 patients (3, 11–15, 19–21), digital clubbing in 3 (11, 12, 21), and fatigue or palpitations in 3 (13, 18, 21). The patient had no prior medical history, had never taken any medications, and had no history of dental caries or tooth extraction, nor had undergone any cardiac surgery. Apart from moderate aortic stenosis with regurgitation discovered upon admission, it was difficult to explain the cause of infection with *S. sinensis*. The initial symptoms were primarily redness, swelling, warmth, pain in the ankle joint, and fever, which resembled those reported by Zhang Y. et al. (17). However, the subsequent development of cerebral infarction and the absence of vegetations on echocardiography within 3 weeks after blood culture positivity for *S. sinensis* remained puzzling, complicating the clinical diagnosis of embolic stroke of cerebral infarction. Moreover, among previously published cases, only one patient presented neurological symptoms (6), who died despite treatment. In contrast, this patient’s main clinical presentation involved cerebral infarction complicated by hemorrhage, significantly increasing the risk of mortality and making therapeutic decisions more challenging. Fortunately, the patient eventually stabilized and was discharged, providing valuable reference for managing patients with infective endocarditis caused by *S. sinensis* complicated by neurological manifestations. On the other hand, it remains unclear whether *S. sinensis* initially infected the joint and then spread to the heart valves, or originated from the heart valves and disseminated via the bloodstream to the joints. Based on the improvement of patient following linezolid therapy, we speculate that dissemination likely occurred from the joint to the heart valve, suggesting that this organism has the potential to affect multiple organ systems.

**Table 2:** Literature review of reported infective endocarditis cases caused by *S. sinensis*.

Number	Authors	Year	Gender	Age	Country	Preexisting conditions	Diagnostic methods	Clinical feature	Valve involvement	Antibiotics and duration	Vegetations	Surgical intervention	Follow-up or outcome
1	Woo et al. (3)	2002	F	42	China	RHD	BC	Fever, a grade 3/6 pansystolic murmur over the cardiac apex radiating to the left axilla, a 30 mm erythematous nodule over the left palm	Mitral regurgitation	Ampicillin for 4 weeks, gentamicin for 2 weeks	No	No	Survived
2	Woo et al. (8)	2004	NA	NA	China	RHD	BC	Fever, finger clubbing	NA	Penicillin + gentamicin	Yes	NA	Survived
3	Uçkay et al. (9)	2007	M	57	Switzerland	RHD and dental surgery	BC	Fever, weight loss, a grade 5/6 proto-mesosystolic murmur	Severe mitral insufficiency	Penicillin G for 3 weeks, gentamicin for 3 weeks, ceftriaxone for 3 weeks	Yes	Yes	Survived
4	Faibis et al. (10)	2008	M	55	France	Carious cavity required tooth extraction treatment	BC	Fatigue, night sweats, weight loss, pansystolic heart murmur of 3–4/6	Severe mitral valve regurgitation	Amoxicillin for 4 weeks, gentamicin for 2 weeks	No	Yes	Survived
5	Seta et al. (11)	2015	F	20	France	RHD	BC	Fever, systolic heart murmur, amygdalitis	Patent ductus arteriosus	Amoxicillin + gentamicin for 4 weeks	Yes	Yes	Survived
6	Goret et al. (12)	2018	M	37	France	Carious cavity required tooth extraction treatment	BC	Fever, weight loss, a grade 3 systolic murmur, purpura of the lower extremities	Massive mitral regurgitation	Amoxicillin for 3 weeks, Gentamicin for 1 week	Yes	Yes	Survived
7	San Francisco et al. (13)	2019	M	63	Caucasian, Travel to Asia	Giant cell arteritis treated with corticosteroids	BC	General malaise, fatigue, temporal headaches	A long diastolic murmur of severe aortic regurgitation	Amoxicillin + gentamicin, penicillin G, ceftriaxone	Yes	Yes	Survived
8	van Ommen et al. (6)	2020	M	58	Netherlands	Severe kidney dysfunction	BC	Fever, Tiredness, weight loss, a holosystolic heart murmur grade 3 of 6 at the apex	Severe mitral insufficiency	Penicillin + gentamicin	Yes	No	Death
9	Zhang et al. (14)	2022	M	19	Mainland China	Bicuspid aortic valve	BC	Fever, numbness and pain in both feet, difficulty squatting, lower extremity edema	Aortic valve bicuspid malformation (Type 0)	Penicillin G + gentamicin, ceftriaxone, teicoplanin + meropenem,	Yes	Yes	Survived
10	Cheng et al. (15)	2023	M	59	China	Coronary arteriography	BC	Palpitation, Tiredness	Regurgitation of the mitral valve (localized), Left ventricular diastolic dysfunction	Meropenem + vancomycin + ceftriaxone	Yes	Yes	Survived
11	Pan et al. (16)	2024	F	40	China, Beijing	Bioprosthetic aortic valve	BC	Fever, a grade 4 holosystolic murmur, an 8-millimeter erythematous tender lesion appeared on the left ring finger	Increased transprosthetic valvular peak velocity	Penicillin G for 2 weeks, ceftriaxone for 4 weeks + amikacin for 2 weeks, teicoplanin & levofloxacin for 2 weeks, meropenem for 2 weeks	Yes	Yes	Survived
12	Manugu et al. (17)	2024	F	8	India	CHD	BC	Fever, chills, cough, shortness of breath	Right ventricular hypertrophy, severe tricuspid regurgitation	Ciprofloxacin + gentamicin	Yes	Yes	Survived
13	Wang et al. (18)	2025	M	33	China, Shanxi	Root canal treatment	BC	Fever, cough, Palpitation, chest distress	Mild to moderate aortic regurgitation, moderate to severe mitral regurgitation	Vancomycin for 4 weeks + linezolid for 2 weeks	Yes	Yes	Survived
14	This article	2025	M	68	China, Sichuan	Aortic Stenosis with Regurgitation	BC	Fever, metatarsophalangeal joint pain, lower extremity edema	Mild to moderate aortic regurgitation	Linezolid + cefazolin for 4 weeks, linezolid + moxifloxacin for 4 weeks	Yes	No	Survived

F, Female; M, Male; RHD, Rheumatic heart disease; BC, Blood culture; NA, Not available.

## 5 Conclusion

*Streptococcus sinensis-*associated IE is rare and often presents insidiously but may result in severe cardiovascular and cerebrovascular complications. Clinicians should maintain a high index of suspicion and promptly perform repeated blood cultures and echocardiographic evaluations in patients with fever of unknown origin accompanied by embolic events. Therapeutic strategies should be individualized based on antimicrobial susceptibility profiles and the patient’s clinical response, with particular attention to antibiotic regimens that achieve adequate tissue penetration. Multidisciplinary management of associated complications is essential to optimize clinical outcomes.

## Data Availability

The original contributions presented in the study are included in the article/Supplementary material, further inquiries can be directed to the corresponding author.
