# Supplementary material for: Infective endocarditis complicated with multiple cerebral embolism and hemorrhage caused by Streptococcus sinensis: a rare case report and literature review
Source: Front Med (Lausanne). 2026 Jun 24;13:1786870. doi: 10.3389/fmed.2026.1786870 (PMC13341527; doi:10.3389/fmed.2026.1786870)
Supplement: Supplementary file 1 [file Table_1.DOCX]

**Supplementary Material**

**Table S1** Results of laboratory testing after admission.

| Indicator | Reference | 1st day | 3rd day | 9th day | 16th day | 19th day | 47th day | 51st day | 56th day | 87th day |
| --- | --- | --- | --- | --- | --- | --- | --- | --- | --- | --- |
| WBC*10^9/L | 3.5-9.5 | 10.15 | 10.55 | 8.21 | 7.23 | 11.96 | 6.84 | 9.3 | 4.82 | 6.05 |
| NEUT%(%) | 40-75 | 78.9 | 76.1 | 73.5 | 75 | 83.4 | 68 | 72.6 | 61.9 | 60.9 |
| CRP mg/L | 0-10 | 76.22 | 76.22 | 42.93 | 53.18 | 67.41 | 44.73 | 109.98 | 19.12 | - |
| PCT ng/mL | 0-0.046 | 0.37 | 0.21 | 0.12 | 0.14 | 0.22 | 0.18 | - | - | - |
| UA umol/L | 208-428 | 320.9 | 264.7 | - | 224.6 | 224.7 | - | 204 | 282.3 | - |

WBC: White blood cell; NEUT%: Percentage of neutrophilic granulocyte; CRP: C-reactive protein; PCT: Procalcitonin; UA: Uric acid.


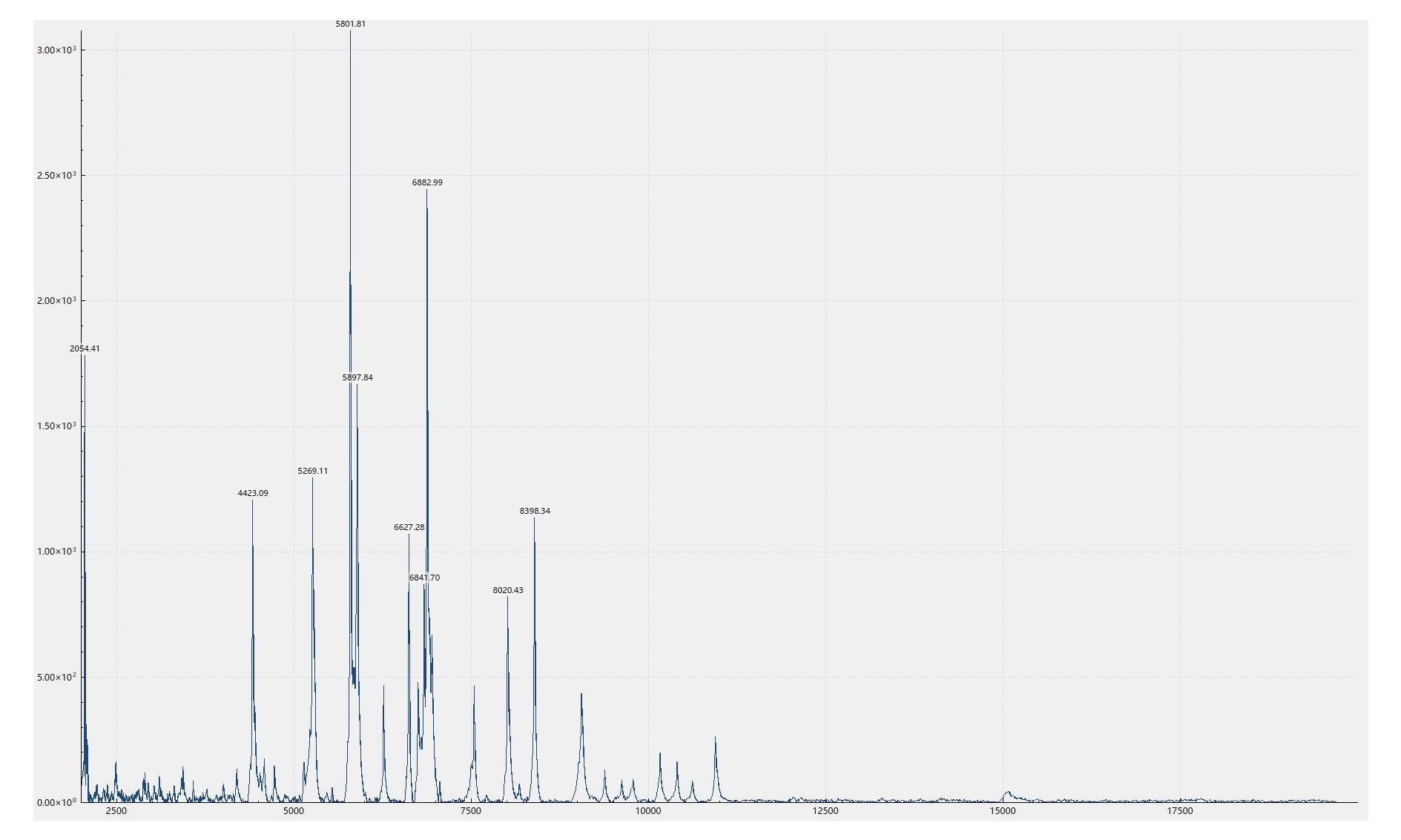


**Figure S1** Mass spectrometry peak patterns of *Streptococcus sinensis* isolates (charge-mass ratio: 2.27). According to the interpretation criteria, identification scores <1.7 indicate unreliable identification, 1.7–2.0 indicate genus-level identification,and ≥2.0 indicate species-level identification
